# Iron protects childhood acute lymphoblastic leukemia cells from methotrexate cytotoxicity

**DOI:** 10.1002/cam4.2982

**Published:** 2020-03-16

**Authors:** Marjan Abedi, Soheila Rahgozar, Abolghasem Esmaeili

**Affiliations:** ^1^ Department of Cell and Molecular biology & Microbiology, Faculty of Biological Science and Technology University of Isfahan Isfahan Iran

**Keywords:** acute lymphoblastic leukemia, drug resistance, iron, methotrexate

## Abstract

Drug resistance is a fundamental clinical concern in pediatric acute lymphoblastic leukemia (pALL), and methotrexate (MTX) is an essential chemotherapy drug administered for the treatment. In the current study, the effect of iron in response to methotrexate and its underlying mechanisms were investigated in pALL cells. CCRF‐CEM and Nalm6 cell lines were selected as T and B‐ALL subtypes. Cells were pretreated with ferric ammonium citrate, exposed to the IC50 concentration of MTX and cell viability was assessed using MTT, colony formation, and flow cytometry assays. Iron‐loaded cells were strongly resistant to MTX cytotoxicity. The inhibitory effect of N‐acetyl cysteine to reverse the acquired MTX resistance was greater than that of the iron chelator, deferasirox, highlighting the importance of iron‐mediated ROS in MTX resistance. Subsequently, the upregulation of *BCL2*, *SOD2*, *NRF2*, and *MRP1* was confirmed using quantitative RT‐PCR. Moreover, a positive correlation was demonstrated between the *MRP1* expression levels and bone marrow iron storage in pALL patients. Further supporting our findings were the hematoxylin and eosin‐stained histological sections showing that iron‐treated nude mice xenografts demonstrated significantly more liver damage than those unexposed to iron. Overall, iron is introduced as a player with a novel role contributing to methotrexate resistance in pALL. Our findings suggest that the patients' bone marrow iron stores are necessary to be assessed during the chemotherapy, and transfusions should be carefully administrated.

## Graphical abstract

Iron a tiny molecule with huge effects. Our findings showed that iron participates in drug resistance and iron‐overload can be considered as a risk factor for relapse. In an attempt to minimize the probability of drug resistance and improve the treatment outcomes in children with ALL, it is suggested that patients' bone marrow iron stores must be assessed during chemotherapy, and the number of blood transfusions should be carefully monitored.

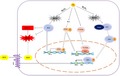

**Abbreviations**
ALL: acute lymphoblastic leukemia
BMIS: bone marrow iron stores
DCFH‐DA: 2′,7′‐Dichloro‐fluorescin diacetate
DFX: deferasirox
FAC: ferric ammonium citrate
MDR: multidrug resistance
MTX: methotrexate
NAC: *N*‐acetyl cysteine
ROS: reactive oxygen species

## 1 INTRODUCTION

Acute lymphoblastic leukemia (ALL) is a blood cancer in which the immature lymphoid progenitors are neoplastic and show deregulated proliferation. This malignancy is the most common type of leukemia in children under 15 years of age.^1^ Despite impressive improvements in the management of ALL, treatment failure still occurs in nearly 20%‐30% of patients.^2^, ^3^ Moreover, the overall survival of patients with relapsed ALL has remained between 25% and 40% over the years.^4^ Although many genetic and epigenetic factors may play role(s) in relapse, multidrug resistance (MDR) is considered as the major element.^5^, ^6^ Several molecular mechanisms are proposed for the generation of MDR, including the upregulation of ABC transporters contributing to a decrease in the cellular accumulation of drugs, mutations in the key genes controlling cell death, and the (hyper) activation of DNA repair, antiapoptotic molecules and survival‐related signaling pathways.^7^

Iron metabolism is shown to be altered in tumors.^8^ This modification can be partially explained by the iron critical impact on cell biology. The biosynthesis of heme, for example, and the production of Fe‐S clusters may regulate the activity of the myriad enzymes modulating cellular metabolism and proliferation.^9^ On the other hand, iron overload is implicated as a carcinogen in some studies.^10^, ^11^, ^12^ The possible mechanisms through which iron may exert its carcinogenic effects include the generation of reactive oxygen species (ROS), activation of the oxidative responsive transcription factors such as AP‐1 and NF‐ĸB, influencing JNK and MAPK pathways, and induction of several alterations in the immune system response and cell cycle growth.^12^, ^13^ Moreover, iron may play a role in drug resistance. An association between tumor chemo‐resistance and H‐ferritin subunit overexpression of iron was previously reported.^14^ Furthermore, it has been revealed that there is a close relationship between iron deprivation and reduced MDR1 expression in the K562 cell line.^15^ Additionally, Li and colleagues demonstrated that iron strengthens the paracrine loop of IL‐6 and confers resistance to breast cancer cells against chemotherapy.^16^ However, the exact molecular mechanism by which this tiny but vital element can affect the response to therapy remains to be fully established.

A positive correlation between the ALL patients bone marrow iron stores and poor response to treatment was previously shown.^17^ Consequently, we hypothesized that iron may participate in drug resistance, and iron overload can be considered a risk factor for ALL relapse. In an attempt to explicit the role of iron in the leukemia cells survival and response to therapy, ferric ammonium citrate was applied on leukemia cell lines and response to methotrexate was primarily investigated. Supportive findings were then obtained through additional ex vivo experiments and generation of ALL xenograft models.

## 2 MATERIAL AND METHODS

### 2.1 Cell lines

The authenticated leukemic cell lines CCRF‐CEM and Nalm6 were obtained from the cell bank of Pasteur Institute of Iran. Cells at passages 10‐25 were used for experiments. The culture medium of these cell lines was RPMI1640 (Gibco) supplemented with 10% heat‐inactivated fetal bovine serum, FBS (Gibco). Cells were incubated in a humidified atmosphere containing 5% CO_2_.

### 2.2 Patients and sampling

Eighteen children referred to the Sayed‐ol‐Shohada Hospital (Isfahan, Iran) and newly diagnosed with Philadelphia negative ALL (5 females, 13 males; mean range, 5.09 ± 0.84 years; range, 0.8‐14 years) entered the study in addition to 15 noncancer age matched controls. The project was conducted in accordance with the Declaration of Helsinki, and permitted by the Ethics Committee of the University of Isfahan (agreement number 89/72763). Bone marrow samples were collected with full written informed consent of parents. Extraction of the mononuclear cells and RNA isolation was performed as previously described.^18^ Primary data obtained in ALL patients analyses are illustrated in Table S1. Response to treatment in the ALL patients was assessed using the presence of minimal residual disease (mrd), a year after treatment. Gene rearrangements of immunoglobulin heavy chain (IgH) and T‐cell receptor gamma (TcRγ) were assessed using PCR‐SSCP (polymerase chain reaction coupled single‐strand conformation polymorphism).

### 2.3 Chemicals and reagents

Ferric ammonium citrate (FAC) was acquired from Merck. Human holo‐transferrin, 2′,7′‐Dichloro‐fluorescin diacetate (DCFH‐DA), *N*‐acetyl‐l‐cysteine (NAC), potassium ferrocyanide and nitric acid 65% were purchased from Sigma. Methotrexate (MTX) was bought from Santa Cruz Biotechnology, Inc. Deferasirox (Exjade) (DFX) was obtained from Novartis.

### 2.4 Viability/proliferation assays

Cellular proliferation capacity and viability were evaluated by MTT assay (Atocel). CCRF‐CEM and Nalm6 cells were seeded into 6‐well plates (1 × 10^6^ cells/well) with exposure to different concentrations of FAC, from 0 to 6400 µmol/L, for 24 hours. Afterwards, cells were washed twice with PBS, then seeded into 96‐well plates (10 × 10^4^ cells/150 µL culture medium) and incubated with the approximate IC50 concentration of MTX (0.5 µmol/L for CCRF‐CEM and 1 µmol/L for Nalm6) for 72 and 96 hours, respectively (in relation to their cell doubling time). Subsequently, 10 µL of MTT solution (0.5 mg/mL) was added into the wells. The formazan crystals were dissolved with 100 µL DMSO after 3 hours, and absorbance was measured using a stat fax 2000 microplate reader (Awareness Technology, Inc) at 492 nm wavelength. All the experiments were carried out at least with three different subcultures of cells and performed in triplicate in each run. To investigate whether there was a direct association between iron and MTX resistance, cells were pretreated with 20 µmol/L DFX for 15 minutes, and 1000 µmol/L NAC for 3 hours, followed by exposure to 400 µmol/L FAC for 24 hours. Cells were then treated with the IC50 concentration of MTX for 72 hours and percent viability was assessed and compared with cells untreated with either DFX or NAC, using MTT assay.

### 2.5 Intracellular iron measurement

The cellular iron uptake was validated by the quantification of the total iron content of cells using atomic absorption flame emission spectrophotometry (AAS). Cells were treated with 400 µmol/L FAC for 24 hours in the 6‐well plates (10 × 10^5^ cells/2 mL culture medium). Cells were then washed twice with PBS, harvested and counted. 30 × 10^5^ cells were suspended in 65% HNO_3_ (1 mL) for 24 hours at room temperature. Finally, lysed cells were analyzed by AA‐6200 Atomic Absorption Spectrophotometer (Shimadzu Scientific Instruments, Inc). The concentration of iron was calculated per 10^6^ cells.

### 2.6 Colony formation assay

To investigate the proliferation capacity of the cells, their ability to form colonies in a semisolid medium was evaluated. The suspended cells (10 × 10^3^ cells/1.5 mL/well) were plated into 6‐well plates over an agar underlay (0.3% agar), in a medium containing 10% FBS, 0.1% penicillin‐streptomycin and 0.5% agar. Cells were incubated in a 5% CO_2_ atmosphere at 37°C for 21 days. Subsequently, 40 µL of MTT solution (5 mg/mL) was added to each well and incubated for 96 hours. Eventually, the number of colonies was counted using a light microscope by three researchers in a blind manner according to Table 1. Finally, the percentage of colony formation efficiency, CFE, was calculated as below.

$$\text{CFE}\%=\frac{\text{Number of colonies in the treated well}}{\text{Number of colonies in the untreated well}}\times 100.$$

**TABLE 1:** The colony counting scoring criteria

Grade	Type of observed colonies	Average of counted colonies in five microscopic fields for each well
1	Colonies with point to small line view	A
2	Colonies with small star‐like shape to small branched line view	B
3	Colonies with intermediate size (two‐ to threefold bigger than the colonies with grade 1)	C
4	Colonies with large size (two‐ to threefold bigger than the colonies with grade 3)	D
Total number of colonies in each well = (A × 1) + (B × 2) + (C × 3) + (D × 4)		

### 2.7 Apoptosis detection by flow cytometry

Cells apoptosis rate was detected by Annexin A5 Apoptosis Detection Kit (Biolegend) according to the manufacturer's guidelines. Briefly, cells (10 × 10^6^ cells/mL) were suspended in Annexin V binding buffer. The tubes containing 100 µL of cell suspension were supplemented by 5 µL FITC Annexin V and 10 µL Propidium Iodide solution. They were then incubated at room temperature for 15 minutes in the dark. 400 µL Annexin V binding buffer was added to each tube and analyzed by flow cytometry.

### 2.8 Detection of the ROS

To detect ROS generation, cells (5 × 10^5^/well) were incubated in 500 µL FBS‐free medium containing 40 μmol/L DCF for 15 minutes in dark. Samples were run in duplicates. Cells were then diluted with 500 µL ice‐cold PBS and the fluorescence intensity was measured, immediately, using a Partec CyFlow ML Flow Cytometer supported by FloMax software in the FL‐1 channel. Histograms were plotted using FlowJo software version 7.6.1. H_2_O_2_ was used as a positive control.

### 2.9 RNA isolation and real‐time PCR

RNA isolation, DNase treatment and cDNA synthesis were carried out using TRIzol reagent (Ambion), DNase I and cDNA synthesis kit (Thermo Scientific) according to the manufacturer's instructions, respectively. Real‐time PCR was performed using SYBR Premix Ex Taq II kit (Takara) according to the previously described method.^18^ All PCR reactions were done in duplicates during two separate experiments using Chromo 4 Real Time PCR Detection System (Bio‐Rad Laboratories, Inc). The comparative 2^−ddCT^ method was used for data analysis and calculation of relative quantification of gene expression. Two separate experiments were performed for each gene. A list of primers is given in Table S2.

### 2.10 SOD activity assay

Total superoxide dismutase activity was assessed according to the standard method described by Beauchamp and Fridovich.^19^ Briefly, 2 × 10^6^ cells were suspended in 1.5 mL extraction buffer, containing 0.01 M phosphate buffer and 0.2% polyvinylpyrrolidone (PVP) (Sigma), and centrifuged at 10 774 *g* for 20 minutes at 4℃. Then, 50 µL of supernatant was added to a 1000 µL reaction solution containing 50 mnol/L potassium phosphate buffer (pH 7.8), 13 mnol/L methionine, 2 mnol/L riboflavin, 75 mnol/L nitroblue tetrazolium (NBT) (Sigma), and 0.1 mnol/L EDTA. Test tubes were incubated at room temperature at 5000 lux light intensity for 15 minutes. Finally, the rate of NBT reduction was spectrophotometrically measured at 560 nm. One unit of superoxide dismutase was assigned as the amount required for 50% inhibition of the NBT reduction. The enzyme activity was calculated as units per µg protein.

### 2.11 Animal model and treatments

Athymic nude mice (C57BL/6 Nude, 4‐ to 6‐week‐old female) were obtained from Pasteur Institute of Iran. Animal experiments were performed in the Department of Biology Specific‐Pathogen‐Free Animal Laboratory, University of Isfahan, and approved by the university's Ethics Committee on Animals Handling (Ethic number: IR.UI.REC.1396.056). Cages, beddings, water, and foods were sterilized and changed twice a week. After a 2‐week delay for giving the animals enough time for adaptation, mice received intraperitoneal administration of 300 mg/kg cyclophosphamide. Seventy‐two hours later, 15 × 10^6^ CCRF‐CEM cells in 100 µL FBS were injected subcutaneously into the mice upper midback. Iron treatment began 3 days after transplantation by injecting 50 mg/kg iron sucrose (Venofer) intraperitoneally twice a week. In order to verify the engraftment, heart blood was analyzed by flow cytometry using antibodies against human CD7, CD3, CD5, and CD4. After 20 days, transplantation was confirmed and chemotherapy was started the day after treatment. 5 mg/kg MTX was inoculated intraperitoneally into each mice, once a week, for 25 days. Mice were sacrificed by deep anesthesia using chloroform in an appropriate chamber, 2 months posttransplantation. Spleen, liver, brain, and bone marrow were harvested after death and were stained with H&E, according to the conventional technique.

### 2.12 Prussian blue staining

For the detection of iron storage levels in patients samples, Perl's Prussian blue staining was carried out. The air‐dried bone marrow specimen was fixed in methanol for 30 s. After alcohol evaporation, staining with the ferrocyanide potassium solution (2 g per 40 mL 3.6% hydrochloric acid) was performed at room temperature for 30 minutes. Slides were counterstained with neutral red (100 mg dissolved in 100 mL ethanol 50%) for 10 minutes and rinsed in tap water. The mounted slides were inspected under a light microscope. Iron grading was performed using the conventional Gale's method.^20^ Murine spleen and liver tissues were fixed in 10% formalin, and then embedded in paraffin. Subsequently, the tissue sections were deparaffinized, hydrated and Prussian blue staining was carried out. Finally, the sections were dehydrated, cleared in xylene and mounted in synthetic resin.

### 2.13 Statistical analysis

All data were presented as the mean ± SEM and the probability value of *P* < .05 was considered to be significant. Differences between the groups were evaluated by using unpaired and two‐tailed *t* tests. The correlation between the patients' bone marrow iron stores and *MRP1* expression levels was determined by chi‐square test. Results were statistically analyzed using Graph Pad Prism 7.0.

## 3 RESULTS

### 3.1 Protective effect of iron in response to MTX

**FIGURE 1:**
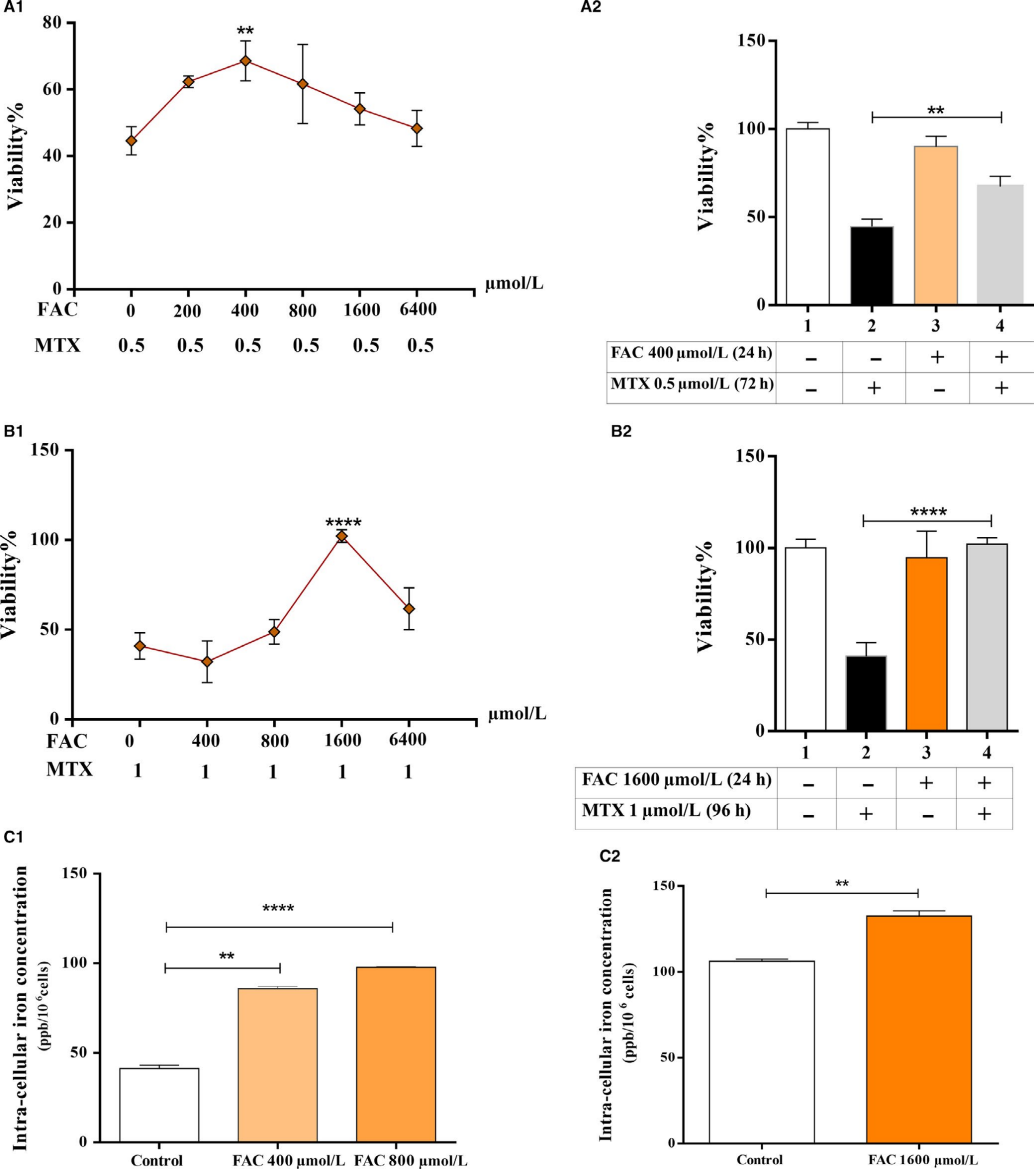
The pretreatment of cells with FAC for 24 h protected cells from MTX cytotoxicity. (A‐1, 2) The CCRF‐CEM cell line was treated with increasing concentrations of FAC (0‐6400 µmol/L) for 24 h. Cells were washed twice with PBS, then seeded and incubated with the approximate IC50 concentration of MTX (0.5 µmol/L) for 72 h. Cell viability was then assessed using MTT assay. Statistical analysis for 400 µmol/L FAC showed maximum protection from MTX. (B‐1, 2) The Nalm6 cell line was treated with increasing concentrations of FAC (0‐6400 µmol/L) for 24 h. Cells were washed twice with PBS, then seeded and incubated with the IC50 concentration of MTX (1 µmol/L) for 96 h. Cell viability was then assessed using MTT assay. Statistical analysis for 1600 µmol/L FAC showed maximum protection from MTX. (C‐1) CCRF‐CEM and (C‐2) Nalm6 cells were treated with FAC for 24 h and lysed by 65% HNO3. The intracellular iron content was then measured per 10^6^ cells using atomic absorption flame emission spectrophotometry (AAS). Results showed a significant increase in intracellular iron upon cells exposure to FAC. Values are mean ± SEM of five separate experiments in triplicates, **P < .01 and ****P < .0001

A positive correlation was previously demonstrated between the bone marrow iron stores of ALL patients and poor response to treatment. Consequently, we hypothesized that iron participates in drug resistance, and iron overload can be considered a risk factor for relapse.^17^ To elucidate the in vitro role of iron in response to therapy, MTX was considered as a key chemotherapy agent in leukemia treatment, and the impact of FAC on MTX‐treated CCRF‐CEM and Nalm6 cells was assessed. Iron‐loaded cells were established by 24 hours pretreatment with FAC. Cells were exposed to the IC50 concentrations of MTX (0.5 and 1 µmol/L) and incubated for 72 and 96 hours, respectively. The difference between the incubation times and the IC50 concentration of MTX for the two cell lines was due to their diverse doubling times and different sensitivities to MTX (data not shown). 400 and 1600 µmol/L FAC showed maximum protection from MTX for CCRF‐CEM and Nalm6 cell lines, respectively (Figure 1A‐1,B‐1).

**FIGURE 2:**
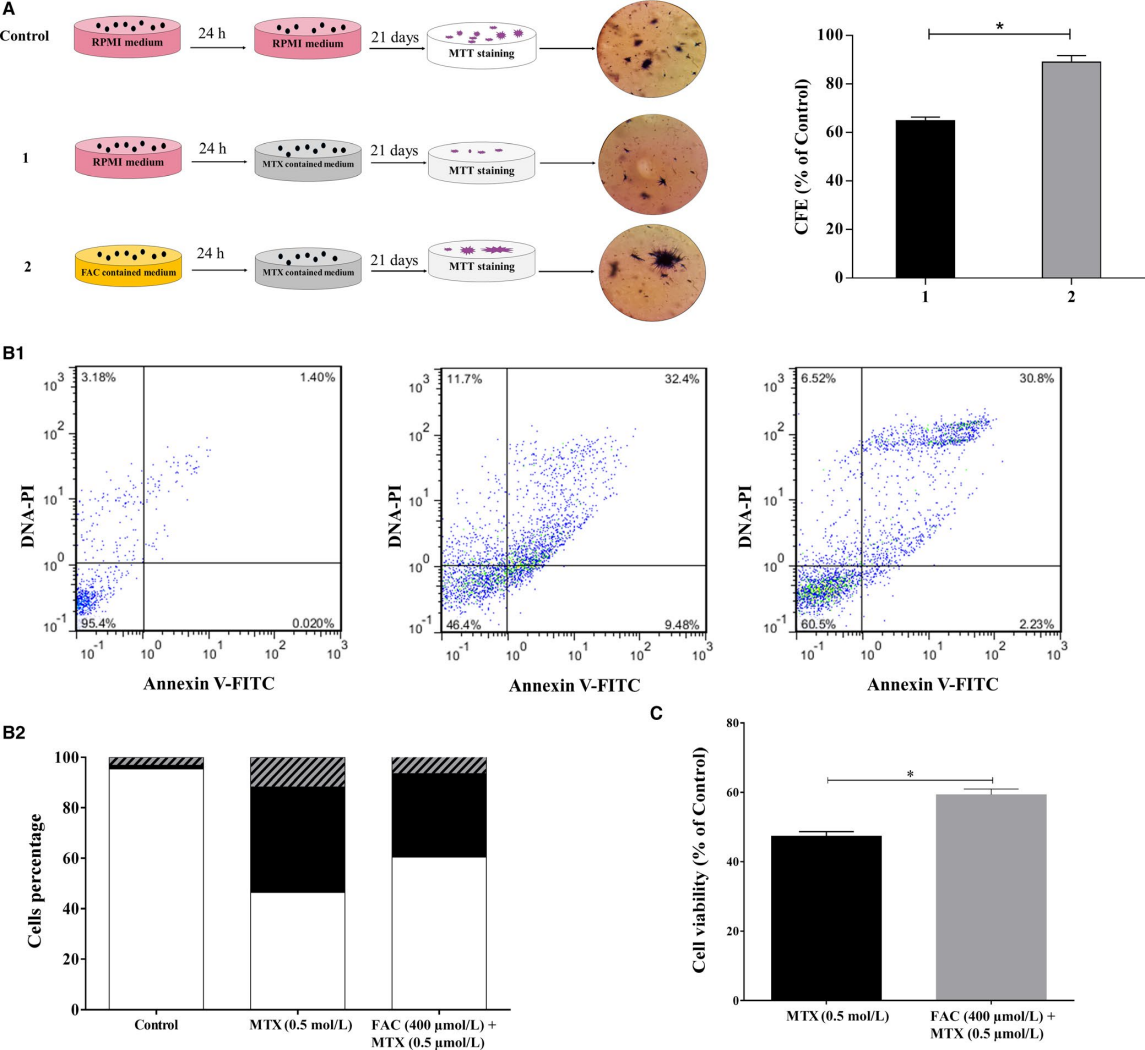
Iron remarkably increased the colony formation efficiency and decreased the percent apoptosis. (A) Colony formation assay was performed by incubation of 24 h FAC pretreated CCRF‐CEM cells with 0.125 µmol/L MTX for 21 d. Colonies were stained by MTT and colony formation efficiency (CFE) was calculated as a percentage of untreated cells (Control). Results showed a pronounced effect on the ability of CCRF‐CEM cells to proliferate and form colonies, in spite of MTX toxicity. (B1) Apoptosis assay was conducted on 24 h FAC pretreated CCRF‐CEM cells followed by 72 h exposure to MTX. It was shown that the rate of apoptosis was decreased in iron‐loaded cells (right panel) compared with the FAC‐untreated samples (middle panel). The left panel shows cells with no treatment, considered as the base line. (B2) The flow cytometry plots shown in 2B1 are illustrated as sectional bars. Solid squares represent apoptotic cells; open squares represent viable cells and dashed squares represent necrotic cells. To calculate the percentage of apoptotic cells, the number of cells shown in the upper right quadrants of the flow cytometry plots were added to the number of the cells demonstrated in the lower right quadrants. Upper and lower left quadrants illustrate the number of necrotic and viable cells, respectively. (C) The rate of viability was significantly increased in 24 h FAC pretreated CCRF‐CEM cells followed by 72 h exposure to MTX compared with the FAC‐untreated samples. Data were shown as percent viability of the untreated control cells. Values are mean ± SEM of two independent experiments in duplicates, *P < .05

Statistical analyses were performed for evaluating the protecting impact of 400 and 1600 µmol/L FAC on CCRF‐CEM and Nalm6 cells, respectively, in the presence of MTX (Figure 1A‐2,B‐2). Results showed considerable resistance to MTX in iron‐loaded cells compared with control cells without iron treatment (67.34 ± 5.83% vs 44.62 ± 4.25% [mean ± SEM; n = 5], *P* < .01 for CCRF‐CEM cells and 102.20 ± 3.49% vs 40.96 ± 7.41% [mean ± SEM; n = 5], *P* < .0001 for Nalm6 cells).

The cellular iron uptake was confirmed by atomic absorption flame emission spectrophotometry prior to MTT assays (Figure 1C).

Further experiments were conducted on CCRF‐CEM cell line since the adaptive resistance to MTX in these cells was generated by lower concentrations of FAC than that of Nalm6.

### 3.2 Colony formation and apoptosis assays

The colony formation ability of 24 hours iron‐loaded CCRF‐CEM cells was studied, while cells were treated with MTX, using soft agar assay. Results showed an increased colony formation efficacy (CFE) of 24.09% for iron‐loaded cells compared with those treated with MTX alone (mean ± SEM; n = 2, *P* = .016) (Figure 2A).

Apoptosis assay was conducted on 24 hours FAC pretreated CCRF‐CEM cells followed by 72 hours exposure to MTX. It was demonstrated that the rate of apoptosis was decreased in iron‐loaded cells compared with the FAC‐untreated samples (33.03% vs 41.88%, respectively). In other words, the rate of viability was increased by 14.10% in those cells (mean ± SEM; n = 2, *P* = .015) (Figure 2B1,2). Data were shown as percent viability of the untreated control cells.

### 3.3 Assessment of the ROS levels in iron‐loaded cells

**FIGURE 3:**
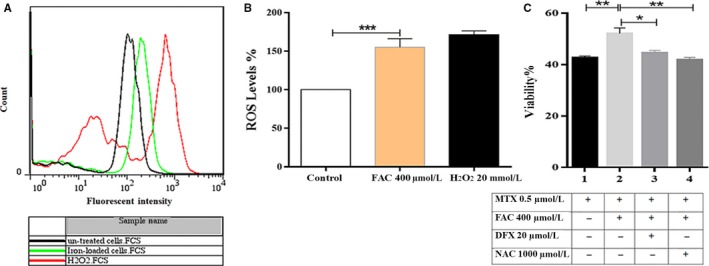
The protective effect of iron against MTX might be, indirectly, through the generation of intracellular ROS. A, ROS detection using 2′,7′‐Dichloro‐fluorescin diacetate (DCFH‐DA). The CCRF‐CEM cells (5 × 10^5^ cells/500 µL per well) were treated with 400 µmol/L FAC, then incubated in FBS‐free medium containing 40 μmol/L DCFH‐DA for 15 minutes in dark followed by dilution with 500 µL ice‐cold PBS. The fluorescence intensity, which was correlated with the amount of the generated oxidized form of the indicator (DCF) and the level of the intracellular ROS, was then measured using flow cytometry. H_2_O_2_ was used as a positive control. The iron‐loaded cells showed a significant shift in fluorescence intensity to the right. B, FAC promoted the intracellular ROS levels up to 55.27 ± 6.36%, compared with the FAC‐untreated cells. C, 15 minutes or 3 h pretreated cells with 20 µmol/L of deferasirox (DFX), an iron chelator, or 1000 μmol/L *N*‐acetyl cysteine (NAC), an ROS scavenger, respectively, were exposed to 400 µmol/L FAC for 24 h. Then, iron‐loaded cells were exposed to the IC50 concentration of MTX for 72 h. Cellular viability was evaluated by MTT assay. Both DFX and NAC counteracted the iron‐induced resistance to MTX by 80.06 ± 0.99% and 109.01 ± 0.96%, respectively. Values are given as the mean ± SEM of two to three separate experiments in triplicates, *P < .05, **P < .01 and ***P < .001

It is widely known that iron produces ROS via Fenton reaction.^21^ The generated ROS levels were particularly higher in iron‐loaded CCRF‐CEM cells than their relative controls (155.27 ± 6.36% vs 100% [mean ± SEM; n = 2], *P* < .001) (Figure 3A,B). This effect was reversed by the addition of deferasirox, an iron chelator, or N‐acetyl cysteine as an ROS scavenger (Figure 3C). Since the impact of N‐acetyl cysteine to reverse the defensive effect of iron against MTX was greater than that of deferasirox, it was suggested that iron might exert its protective effect indirectly, by the generation of intracellular ROS.

### 3.4 Evaluation of the gene expression profiles involved in iron‐induced ROS using real‐time PCR

**FIGURE 4:**
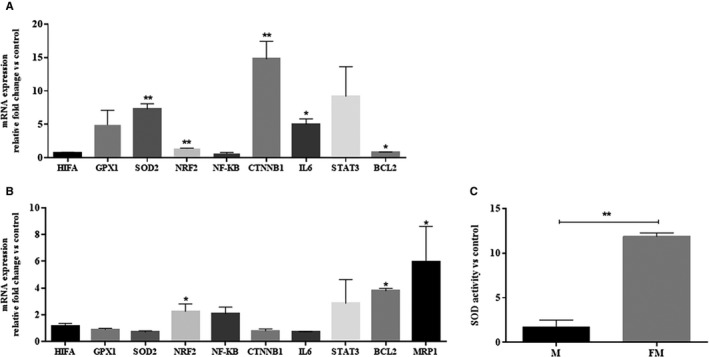
Iron‐induced alterations in the mRNA expression profiles of some iron and ROS related genes. A, The expression pattern of the antioxidant and survival/proliferation‐related genes was measured followed by 24 h treatment of CCRF‐CEM cell line with 400 µmol/L FAC, and compared with those in the untreated cell line. B, The gene transcripts expression profile was assessed in the FAC‐treated cells which were exposed to MTX for 72 h, and compared with that in the iron‐loaded cells without MTX posttreatment. *GAPDH1* was applied as the reference gene. The comparative 2^−ddCT^ method was used for data analysis and the related groups were compared using *t* test. C, The activity of superoxide dismutase was increased by 7.02‐fold in the iron‐loaded cells exposed to 0.5 µmol/L MTX (FM) for 72 h when compared with MTX‐treated cells (M). These data showed both the transcriptional and, indirectly, translational overexpression of the SOD gene upon 24 h exposure to FAC. All values are results of two different experiments in duplicates shown as mean ± SEM, *P < .05 and **P < .01

To unravel the mechanism through which iron may cause resistance to MTX, the impact of FAC on the expression profile of two categories of genes, antioxidants and survival/proliferation‐related genes was assessed using real‐time PCR in CCRF‐CEM cells. It was shown that among the aforementioned genes, the expression levels of *SOD2*, *NRF2*, *CTNNB1 (β‐catenin)*, *IL6*, and *BCL2* were increased in the iron‐loaded cells compared with the FAC‐untreated controls (7.32 ± 0.77, 1.41 ± 0.03, 14.79 ± 2.63, 5.02 ± 0.79, and 0.85 ± 0.03 fold change, respectively) (Figure 4A). Moreover, the expression levels of *NRF2* and *BCL2* remained elevated when cells were incubated with MTX for 72 hours, highlighting the role of these genes in iron‐induced resistance to MTX (2.25 ± 0.56, 3.78 ± 0.19) (Figure 4B). Furthermore, it was demonstrated that the expression level of *MRP1*, the NRF2‐target gene, was increased followed by treatment with MTX by 5.95 ± 2.65 fold (mean ± SEM, *P* < .05). These data may confirm the activation of the transcription factor NRF2 followed by its overexpression. Furthermore, results demonstrated that the activity of superoxide dismutase was increased by 7.02‐fold while treating cells with 400 µmol/L FAC for 24 hours, then 0.5 µmol/L MTX for 72 hours (Figure 4C). These data showed both the transcriptional and, indirectly, translational overexpression of the *SOD* gene upon 24 hours exposure to FAC.

### 3.5 Ex vivo analyses

**FIGURE 5:**
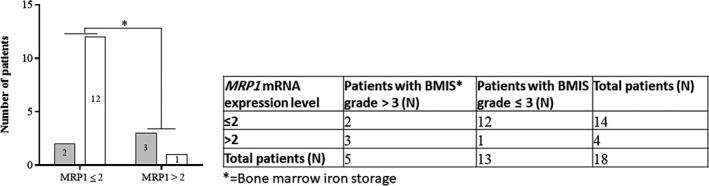
The positive correlation between *MRP1* expression and iron storage in bone marrow. The *MRP1* mRNA expression level of the mononuclear cells, obtained from 18 ALL patients, was associated with their bone marrow iron stores. Real‐time PCR was performed in two different experiments in duplicates to assess the patients cellular *MRP1* expression levels. *GAPDH1* was applied as the reference gene and the comparative 2^−ddCT^ method was used for data analysis. Patients iron stores was graded by Perl's Prussian blue staining, as described in materials and methods. The comparative analysis between the MRP1 expression levels and iron storage was performed using the two‐tailed chi‐square test. Black bars display iron grade > 3, gray bars represent iron grade ≤ 3, BM, bone marrow, *P = .016

To validate the in vitro findings, the expression levels of *MRP1* were quantified in the mononuclear cells harvested from patients with ALL using real‐time PCR. The rationale for selecting MRP1 among other genes, besides its overexpression upon exposure to MTX, was the functional effect of this gene as a drug efflux pump and its correlation with NRF2, as its transcription factor. NRF2 was overexpressed while the cells were overloaded with iron in 24 hours and could have induced the overexpression of MRP1 while cells were subsequently exposed to MTX for 72 hours. The cut‐off point of the *MRP1* expression levels was defined as two. Interestingly, the mRNA expression levels of *MRP1* showed a positive association with the patients' bone marrow iron stores (Figure 5). Results showed that from the three samples with high expression levels of *MRP1* (>2) and iron grade > 3 (Table S1), all patients were low responders to chemotherapy (Table S1). Response to treatment was evaluated by assessing the MRD status of the ALL patients, 1 year after chemotherapy.

### 3.6 In vivo experiments

**FIGURE 6:**
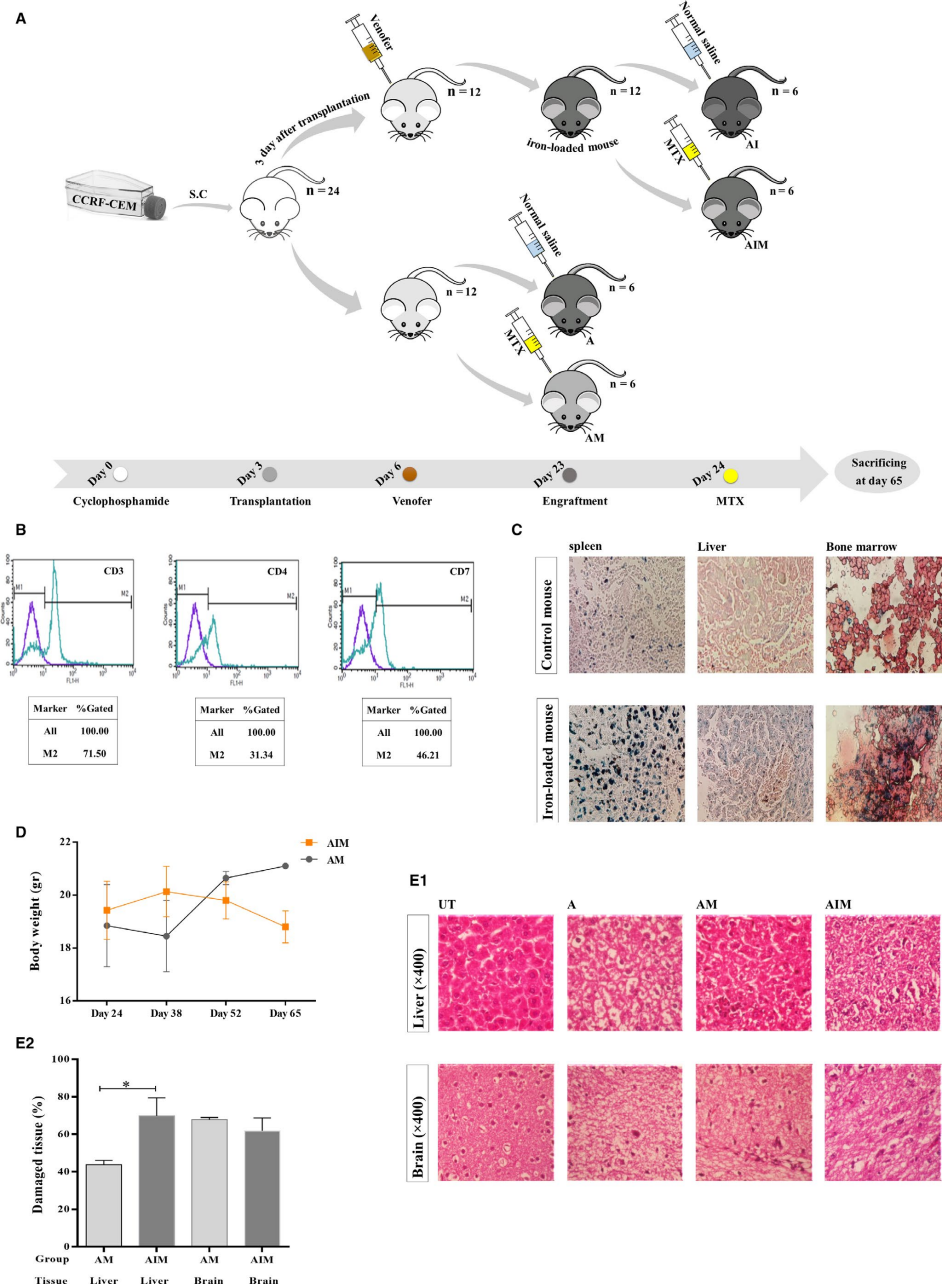
The in vivo experiments. A, Schematic illustration of the in vivo experiments. The timeline of treatments and alleged groups are displayed. C57BL/6 Nude mice received 300 mg/kg intraperitoneal cyclophosphamide, on day 0. 15 × 10^6^ CCRF‐CEM cells were injected subcutaneously in the mice upper midback on day 3. Iron treatments were begun 3 d after transplantation by injecting 50 mg/kg Venofer (iron sucrose) intraperitoneally twice a week. The mice of indicated groups, AIM and AM, received 5 mg/kg intraperitoneal MTX, weekly, the day after the engraftment. Mice were sacrificed 2 mo after transplantation. The control group with no transplantation, is not shown. B, Verification of the leukemia engraftment in mice. A representative flow cytometry‐based detection of the transplanted CCRF‐CEM in the mouse heart blood, using antibodies against human CCRF‐CEM surface markers. The identified human CD3, CD4, and CD7 markers (71.50%, 51.34%, and 46.21%, respectively) confirmed leukemia engraftment. C, The confirmed accumulation of iron in mouse tissues. Representative macroscopic views of spleen, liver, and bone marrow aspirate of the CCRF‐CEM transplant mice, stained by Perl's Prussian blue (×400). Blue dots were significantly increased in the iron sucrose injected tissues compared with those in the control mouse. D, A comparison between the body weights of MTX‐treated iron‐loaded (AIM) and control leukemic mice (AM) from the beginning of the chemotherapy treatment until the last day of experiment. E‐1, Representative microscopic views of liver and brain sections of the abovementioned mice groups, stained with Hematoxylin and Eosin. The iron‐loaded leukemic mice group treated with MTX (AIM) showed significant liver, but not brain damage compared with the control groups of leukemic mice unexposed to iron, but treated with MTX (AM). Significant histological modifications in brain and liver tissues are demonstrated in untreated leukemic mice (A) compared with the un‐transplanted nonleukemic mice with no treatment (UT). E‐2, For quantitative morphometry of liver sections, images were photographed from each stained section using a magnification of 40× objective lens. The images were transformed into RGB stack format via Image J software and the area percent with red staining equal to or greater than a defined threshold was computed with this software. By subtracting the total red‐colored area from 100, the percentage of the damage in each tissue section was calculated. White spaces of the lumens of blood vessels and artifacts were excluded beforehand. Interestingly, results obtained from the blind interpretation of data were consistent with the scoring analysis performed by the Image J program. Three sections per mouse were evaluated. Data were reported as mean ± SEM; *P = .04. A, CCRF‐CEM transplanted leukemic mice; AM, MTX‐treated leukemic mice; AI, iron‐loaded leukemic mice; AIM, iron‐loaded leukemic mice, treated with MTX, UT, Un‐transplanted mice with no treatment

To further validate our findings, in vivo studies were conducted. After a 2‐week delay for giving the animals enough time for adaptation, treatments were started as described in Figure 6A. Iron treatment was initiated at day 3, followed by CCRF‐CEM transplantation, during which 50 mg/kg of Venofer (iron sucrose) was injected twice a week. Iron storage was identified in the mice liver and spleen after three injections, on day 10, at the total iron dose of 150 mg/kg. However, to maintain an iron overload condition in mice, Venofer was persistently administrated up to 850 mg/kg. Perl's Prussian blue staining confirmed the establishment of iron overload mice models (Figure 6C). To identify the in vivo effect of iron on MTX resistance, treatment was initiated the day after the leukemia engraftment was authenticated by flow cytometry (Figure 6B). MTX was injected intraperitoneally, once per week. Mice were sacrificed 2 mo/62 d after transplantation. Iron treatment decreased the survival rate of the ALL mice models by 8.40%. Results were supportive of our in vitro findings, especially the colony formation assay. Effect of iron on the MTX‐treated leukemic mice showed that the CCRF‐CEM transplanted iron‐loaded mice treated with MTX (AIM) group were considerably less responsive to chemotherapy than those unexposed to iron (AM). Altered liver histopathology and decline in body weight were observed in the AIM group by 26.11% and 11%, respectively (Figure 6D,E‐2). These findings strengthened the in vitro results regarding iron involvement in MTX resistance.

## 4 DISCUSSION

Iron plays critical roles in cellular proliferation and metabolic activities.^9^ Several studies have reported its protective role, especially, against cancer.^22^, ^23^, ^24^ In contrast, it has been demonstrated that iron overload may be carcinogenic,^10^, ^25^ contributing to altered cellular metabolism shown in tumors.^8^, ^12^ Moreover, iron is suggested to play a role in drug resistance.^14^, ^15^, ^16^ Given the inconsistency and lack of adequate information regarding the role of iron in ALL, the impetus of this work was to evaluate the impact of this compound on the response of lymphoblastic, malignant cells to MTX and to unveil the molecular mechanism underlying this effect.

Iron was previously introduced as one of the responsible mediators for apoptosis, necrosis, and ferroptosis (iron‐mediated programed cell death).^26^, ^27^, ^28^, ^29^, ^30^ Furthermore, an association between tumor chemo‐resistance and altered intracellular iron content was formerly described.^14^, ^15^ Iron chelation was shown to have antiproliferative effects in several cancers 31, 32, 33 and it was demonstrated that iron‐chelating agents can reverse the resistance of cancer cells to chemotherapeutic agents.^34^, ^35^ Despite emerging interest in using iron chelators in combinational chemotherapy, it has been revealed that some of these reagents, irrespective of their harmful effects on normal cells,^36^ exert their antitumor activity regardless of their chelating function.^37^, ^38^ Ultimately, the mechanism by which iron confers drug resistance to cancer cells has remained elusive. Growing efforts were made to unravel these mechanisms, particularly in breast cancer.^8^, ^16^, ^39^ Considering the effect of iron in ALL, we previously reported a compelling positive correlation between the bone marrow iron stores (BMIS) of ALL patients and poor response to treatment.^17^ The current work has introduced iron, for the first time, as a defender of lymphoblastic malignant cells against MTX cytotoxicity (Figures 1 and 2). The maximum relative adaptive resistance to MTX was induced by the application of 400 and 1600 µmol/L FAC to CCRF‐CEM and Nalm6 cells, respectively. According to the review literature, different concentrations of FAC used for the generation of iron‐loaded cell lines were 360 M for rat heart blood cells,^40^ 30 µmol/L for Hep3B,^41^ and 200 µmol/L for MCF7 cells.^42^ Furthermore, the intracellular iron status of CCRF‐CEM and Nalm6 was different (41.20 ppb vs 106.18 ppb per 1 × 10^6^ cells, respectively) (Figure 1C). It is assumed that this can explain the contrasting sensitivity of these two cell lines to MTX (the IC50 concentrations of 0.5 and 1 µmol/L for CCRF‐CEM and Nalm6, respectively). The intracellular availability of higher concentrations of iron in Nalm6 might be a determinant for its lower sensitivity to MTX. The contribution of different intracellular iron contents in dissimilar responses to H_2_O_2_ was reported in mouse lymphoma cell lines^43^; however, no publication concerning leukemia cells is available. Since the adaptive resistance to MTX in CCRF‐CEM cells was generated by lower concentrations of FAC than that of Nalm6, the remaining experiments were conducted on this cell line.

**FIGURE 7:**
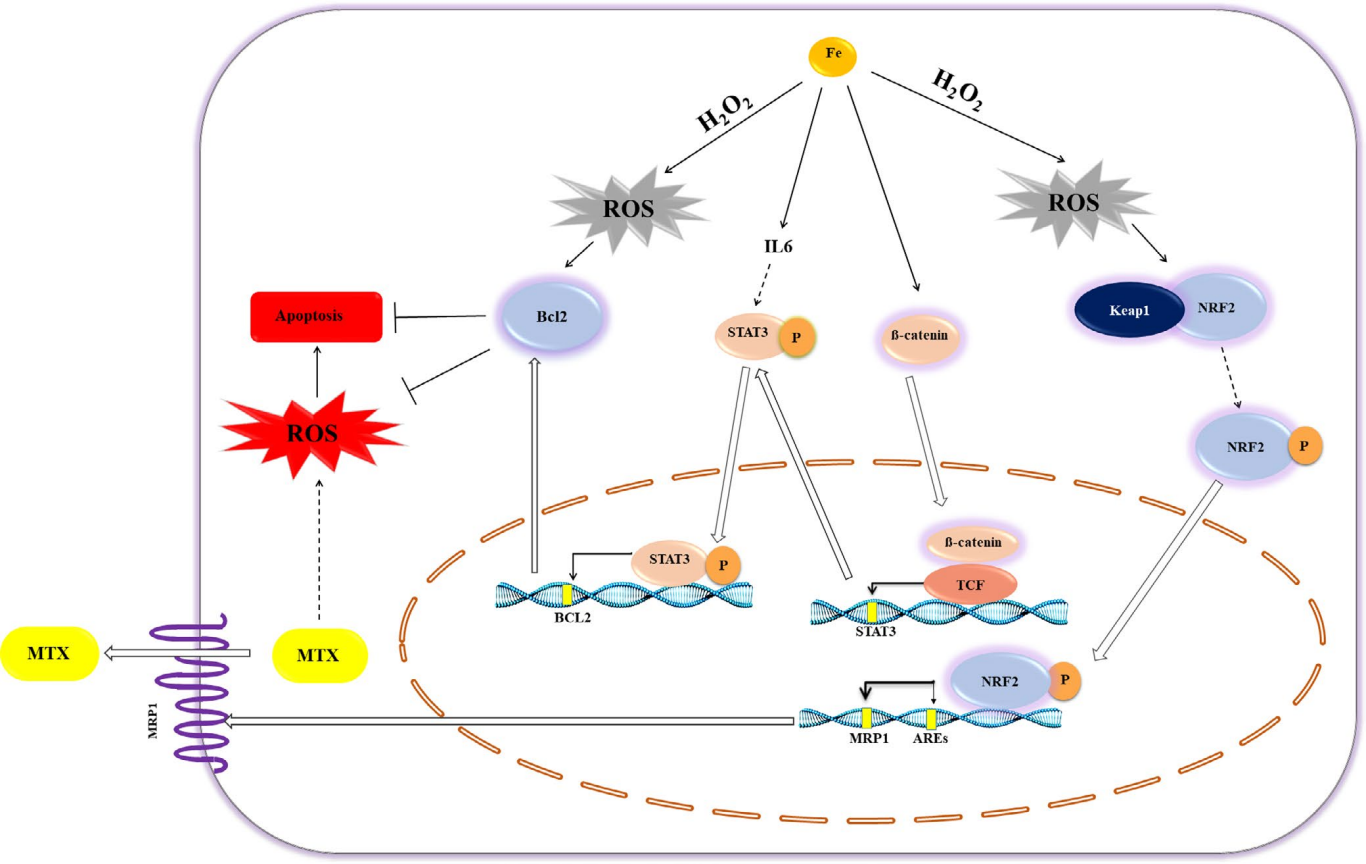
Iron a tiny molecule with huge effects. The schematic diagram demonstrating at least two pathways by which iron may confer resistance to leukemic cells. First, iron induces moderate levels of ROS, shown in gray, through the Fenton reaction mediated by hydrogen peroxide (Fe^2+^ + H_2_O_2_ → Fe^3+^ + OH^−^ + ºOH). ROS, then, may trigger the antioxidant defense system via upregulation of *BCL2* and *NRF2*. Thereby, the malignant cell can survive against the toxic levels of MTX‐induced ROS (demonstrated in red). At the same time, the antiapoptotic activity of *BCL2* may inhibit the MTX cytotoxic effects. Upregulation of *BCL2* may also be mediated by iron itself through the overexpression of *STAT3*, possibly via IL‐6 and β‐catenin. Moreover, iron enhances *MRP1* expression indirectly via the activation of its transcription factor, NRF2. The MTX efflux through this ABC transporter protects the cell from MTX cytotoxicity. 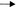, activation; 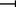, inhibition/repression; 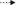, possible/indirect activation; 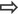, transport; 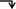, activates transcription of the gene (shown by yellow rectangles); 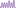, MRP1 transporter; NRF2, nuclear factor erythroid 2‐related factor 2; MRP1, multidrug resistance‐associated protein 1 (also known as ABCC1); TCF, T‐cell factor/lymphoid enhancing factors family of DNA‐binding factors; AREs, antioxidant response elements; RR, ribonucleotide reductase; MTX, methotrexate; ROS, reactive oxygen species

In an attempt to unravel the underlying mechanism(s) contributing to the iron‐mediated resistance against MTX, the present study demonstrated that *BCL2* and *IL‐6* were overexpressed in the iron‐loaded leukemic cells (Figure 4). The developed resistance to MTX and decreased apoptosis in the iron‐loaded ALL cells, in line with the prolonged overexpression of *BCL2*, elicit a binary function for iron as an antiapoptotic and antioxidant molecule in these cells. Moreover, regarding the partial effect of iron on apoptosis (Figure 2B), it is assumed that the antioxidant capacity of this element is more important. The overexpression of *IL‐6* in iron‐loaded cells is consistent with Li and colleagues' investigations into breast cancer.^16^ It is widely known that IL‐6 can activate the STAT3 signaling pathway. STAT3 promotes cell survival and proliferation via upregulation of its target genes, *BCL2* and *c‐myc*.^44^ Although in the iron‐loaded leukemic cells, STAT3 activator, *IL‐6*, and its downstream target, *BCL2*, were both overexpressed, a slight increase in the mRNA expression level of STAT3 was detected. Western blot analysis is required to validate the implication of the STAT3 pathway in the resistance of ALL cells to MTX. Moreover, it is interesting to note that *β‐catenin* mRNA levels were also increased in the iron‐loaded ALL cell line. The positive regulatory role of iron for ß‐catenin is previously described in the colorectal cancer cell lines.^45^ Likewise, it is discovered that the STAT3 promoter possesses a functional element for TCF binding,^46^ through which ß‐Catenin may upregulate the expression of STAT3 in esophageal squamous cell carcinoma. A putative crosstalk between the aforementioned genes is illustrated in Figure 7.

The current work introduces iron, for the first time, as an ROS inducer in ALL cells. Data showed that the antioxidant ability of N‐acetyl cysteine to reverse the defensive effect of iron against MTX was surprisingly greater than that of the iron chelator, deferasirox (Figure 3); implying that iron mainly confers MTX resistance through the induction of intracellular ROS. Support for this hypothesis is our results demonstrating that ROS levels were particularly higher in iron‐loaded cells than their relative control cells. It is interesting to note that iron may promote the intracellular levels of ROS at a critical concentration. It means that the above‐optimal concentrations of FAC do not increase ROS levels relatively (Figure S1). It is assumed that the extra amount of iron may be converted to the antioxidant ferritin inside the cell, thereby inhibiting the intracellular free radicals cytoxicity. Published data showed that iron may indirectly induce the generation of ROS through the hydrogen peroxide participating in the Fenton reaction 21 (Figure 7). Subsequently, the current study illustrated that the pretreatment of CCRF‐CEM cells with FAC and MTX develops upregulation of *SOD2* and *NRF2* genes (Figure 4). It is proposed that the iron‐mediated ROS triggers resetting of the redox state through the overexpression of its two aforementioned key regulators. Although the noteworthy alteration in SOD levels has been described in malignant cells,^47^ its involvement in tumor progression and chemo‐resistance is still controversial.^48^, ^49^, ^50^, ^51^, ^52^ Results demonstrated that the mRNA expression levels of *SOD2* and its enzymatic activity (Figure 4) were increased in the iron‐loaded cells when exposed to MTX. On the other hand, although several attempts have been made to identify the role of *NRF2* in drug resistance,^53^, ^54^, ^55^ our findings were the first data to illustrate the overexpression of this gene in the iron‐loaded ALL cells. NRF2 is a key regulator for both the antioxidant protection and detoxification of cells. Considering the detoxification role of NRF2, it was shown that *MRP1*, the NRF2 target gene and drug efflux pump, is overexpressed in the MTX‐resistant iron‐loaded cell lines as well as the ALL patients' primary cells with high storage of bone marrow iron (Figures 4 and 5). More interestingly, these patients were all poor responders to chemotherapy. Investigating larger populations of ALL patients in prospective cohort studies may help intensify the validity of results provided in this study.

To examine the in vivo relevance of our findings regarding the effect of iron on response to MTX, iron‐loaded leukemic mice were established (Figure 6C). Results showed that despite the acceptable efficacy of MTX treatment in leukemic mice, iron‐loaded animals had notably altered liver histopathology and decline in body weight compared with the leukemic mice unexposed to iron (Figure 6). These data supported the contribution of iron to the poor response to MTX.

Taken together, we introduced iron, for the first time, as a responsible compound with critical implications in the resistance of ALL malignant cells to MTX. ROS acts as a secondary messenger for the iron‐mediated resistance to MTX; and *BCL2*, *SOD2*, and *NRF2* genes are the effector mediators in this scenario. The comprehensive functions of iron in ALL drug resistance have yet to be understood. Additional experiments of RNA interference to knock down the selected mRNAs and Western blot analyses for protein studies must be performed, and further preclinical investigations are required in this burgeoning area of research. However, in an attempt to minimize the probability of drug resistance and improve the treatment outcomes in children with ALL, our findings suggest that the patients' bone marrow iron stores must be assessed during the chemotherapy, and the number of blood transfusions should be carefully monitored.

## CONFLICT OF INTEREST

We have no competing interests to declare.

## AUTHOR CONTRIBUTIONS

Dr Rahgozar had full access to all of the data in the study and takes responsibility for the integrity of the data and the accuracy of the data analysis and was involved in study design. Abedi was involved in acquisition of data and statistical analysis. Abedi, Rahgozar, and Esmaeili were involved in analysis and interpretation of data. Abedi and Rahgozar were involved in manuscript preparation.

## Supporting information

Fig S1

Table S1‐S2

## Data Availability

The data that support the findings of this study are available on request from the corresponding author. The data are not publicly available due to privacy or ethical restrictions.
