# Supplementary figures and images for: Iron protects childhood acute lymphoblastic leukemia cells from methotrexate cytotoxicity
Source: Cancer Med. 2020 Mar 16;9(10):3537–50. doi: 10.1002/cam4.2982 (PMC7221302; doi:10.1002/cam4.2982)

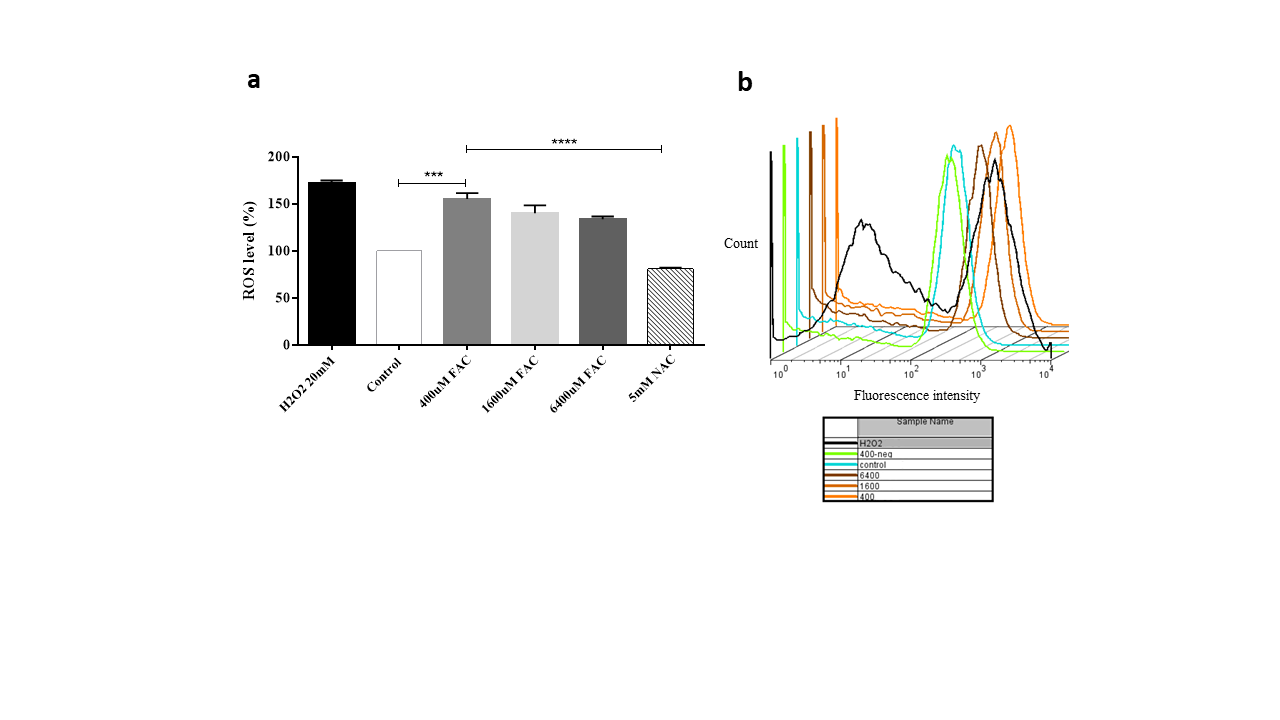

Supplement: Supplementary file 1 — Fig S1 [file CAM4-9-3537-s001.tif]
