# Supplementary material for: Iron protects childhood acute lymphoblastic leukemia cells from methotrexate cytotoxicity
Source: Cancer Med. 2020 Mar 16;9(10):3537–50. doi: 10.1002/cam4.2982 (PMC7221302; doi:10.1002/cam4.2982)
Supplement: Supplementary file 2 — Table S1‐S2 [file CAM4-9-3537-s002.docx]

**Supplementary Figure and tables**

**Figure S1: Iron induces intra-cellular ROS.** **a**) CCRF-CEM cells (5×10^5^ cells/500µl per well) were treated with increasing concentrations of FAC for 24h. Subsequently, the intra-cellular ROS content was detected using 2′,7′-Dichloro-fluorescin diacetate (DCFH-DA). 400µM of FAC significantly enhanced the intra-cellular ROS levels up to 55.27±6.36%, compared with the FAC-untreated cells. However, at higher concentration of FAC, 1600µM and 6400µM, this effect was less than 400 µM (40.5±3.6% and 33.9±2.8%, respectively). b) The iron-loaded cells showed a significant shift in fluorescence intensity to the right. Values are reported as the mean ± SEM of three separate experiments in duplicates, *** = *P*<0.001 and ****= *P*<0.0001

**Table S1. Primary data obtained for ALL patients analyses**

| Patient number | ALL immunophenotype | Patient Sex | Age  (year) | WBC >20 000  (cells/µl) | 2^-ddCt^ of MRP1 expression | Iron Grading* | mrd status** |
| --- | --- | --- | --- | --- | --- | --- | --- |
| 1 | B-cell | M | 7 | - | 3.85455 | 4 | + |
| 2 | B-cell | M | 11 | + | 5.302095 | 6.5 | + |
| 3 | B-cell | F | 7 | - | 2.525489 | 4 | + |
| 4 | T-cell | M | 14 | - | 4.262094 | 3 | + |
| 5 | B-cell | M | 3 | - | 1.626265 | 1 | - |
| 6 | Burkitt | M | 9 | + | 1.527913 | 1 | + |
| 7 | B-cell | F | 3 | - | 1.648967 | 4 | - |
| 8 | T-cell | M | 2 | + | 0.542074 | 1 | - |
| 9 | B-cell | M | 3 | + | 0.134582 | 1 | - |
| 10 | B-cell | M | 7 | + | 0.2855 | 2 | - |
| 11 | B-cell | F | 3 | + | 0.287486 | 1 | - |
| 12 | T-cell | M | 4 | + | 0.419448 | 1 | - |
| 13 | B-cell | F | 3 | - | 0.21562 | 4 | - |
| 14 | B-cell | M | 0.8 | + | 0.220151 | 1 | - |
| 15 | T-cell | M | 3 | - | 0.1991 | 1 | - |
| 16 | B-cell | F | 6 | + | 0.204697 | 3 | - |
| 17 | B-cell | M | 5 | - | 0.094835 | 3 | - |
| 18 | B-cell | M | 0.9 | + | 0.87452 | 3 | + |

*Iron grading was performed as previously described (17)

**mrd=minimal residual disease obtained from RT-PCR assessment one year after chemotherapy

**Table S2. Primer sequences of the interest genes**

| Gene name | HGNC ID | Primer sequence (5′to 3′) | Primer length (bp) | Amplicon length (bp) |
| --- | --- | --- | --- | --- |
| *GAPDH* | HGNC:4141 | F:CCCCAGCAAGAGCACAAGAGGAAGA | 26 | 105 |
|  |  | R:CATGGCAACTGTGAGGAGGGGAGAT | 26 |  |
| *MRP1/ABCC1* | HGNC:51 | F:CGGATGTCATCTGAAATGGGA | 21 | 103 |
|  |  | R:GAGCTGTCTCCTGGATTTGC | 20 |  |
| *IL6* | HGNC:6018 | F: AGGAGACTTGCCTGGTGAAA | 20 | 180 |
|  |  | R: CAGGGGTGGTTATTGCATCT | 20 |  |
| *STAT3* | HGNC:11364 | F: TGAGACTTGGGCTTACCATTGGGT | 24 | 174 |
|  |  | R: TCTTTAATGGGCCACAACAGGGCT | 24 |  |
| *GPX1* | HGNC:4553 | F: CAGTCGGTGTATGCCTTCTCG | 21 | 105 |
|  |  | R: GAGGGACGCCACATTCTCG | 19 |  |
| *CDH1* | HGNC:1748 | F: CCCATCAGCTGCCCAGAAAATGAA | 24 | 175 |
|  |  | R: CTGTCACCTTCAGCCATCCTGTTT | 24 |  |
| *CTNB1* | HGNC:2514 | F: GCAGAGTGCTGAAGGTGCTA | 20 | 94 |
|  |  | R: TCTGTCAGGTGAAGTCCTAAAGC | 23 |  |
| *SOD2* | HGNC:11180 | F: GGGATGCCTTTCTAGTCCTATTC | 23 | 114 |
|  |  | R: TATAGAAAGCCGAGTGTTTCCC | 22 |  |
| *HIF1A* | HGNC:4910 | F: GAGATGTTAGCTCCCTATATCCCA | 24 | 154 |
|  |  | R: TAGGTTCTTGTATTTGAGTCTGCTG | 25 |  |
| *NRF2* | HGNC:7782 | F: CAGCGACGGAAAGAGTATGA | 20 | 200 |
|  |  | R: TGGGCAACCTGGGAGTAG | 18 |  |
| *NFKB* | [HGNC:9955](https://www.genenames.org/data/gene-symbol-report/#!/hgnc_id/HGNC:9955) | F: ATCCCATCTTTGACAATCGTGC | 22 | 153 |
|  |  | R: CTGGTCCCGTGAAATACACCTC | 22 |  |
| *BCL2* | HGNC:990 | F:GGTGAACTGGGGGAGGATTGT | 21 | 232 |
|  |  | R:CTTCAGAGACAGCCAGGAGAA | 21 |  |
